# Supplementary material for: Insights into the modulation of bacterial NADase activity by phage proteins
Source: Nat Commun. 2024 Mar 27;15:2692. doi: 10.1038/s41467-024-47030-z (PMC10973363; doi:10.1038/s41467-024-47030-z)
Supplement: Supplementary file 1 — Supplementary Information [file 41467_2024_47030_MOESM1_ESM.pdf]

Supplementary information

Insights into the modulation of bacterial NADase activity by phage proteins

Hang Yin[1,2#], Xuzichao Li[1,3#], Xiaoshen Wang[1,3#], Chendi Zhang[4#], Jiaqi Gao[1,3], Guimei Yu[1], Qiuqiu He[1,3], Jie Yang[1], Xiang Liu[5], Yong Wei[6]*, Zhuang Li[4]*, Heng Zhang[1,3]*

1. State Key Laboratory of Experimental Hematology, Key Laboratory of Immune Microenvironment and Disease (Ministry of Education), The Province and Ministry Co-sponsored Collaborative Innovation Center for Medical Epigenetics, International Joint Laboratory of Ocular Diseases (Ministry of Education), Tianjin Key Laboratory of Ocular Trauma, School of Basic Medical Sciences, Tianjin Medical University, Tianjin 300070, China.

2. Department of Pharmacology, School of Basic Medical Sciences, Tianjin Medical University, Tianjin 300070, China.

3. Department of Biochemistry and Molecular Biology, School of Basic Medical Sciences, Tianjin Medical University, Tianjin 300070, China.

4. State Key Laboratory of Biocatalysis and Enzyme Engineering, School of Life Sciences, Hubei University, Wuhan 430062, China

5. State Key Laboratory of Medicinal Chemical Biology, Frontiers Science Center for Cell Responses, College of Life Sciences, Nankai University, Tianjin, China.

6. The Cancer Hospital of the University of Chinese Academy of Sciences (Zhejiang Cancer Hospital), Institute of Basic Medicine and Cancer (IBMC), Chinese Academy of Sciences, Hangzhou 310022, China

#These authors contributed equally: Hang Yin, Xuzichao Li, Xiaoshen Wang and Chendi Zhang

Correspondence: weiyong@ibmc.ac.cn (Y.W.), zhuangli@hubu.edu.cn (Z.L.), zhangheng134@gmail.com (H.Z.)

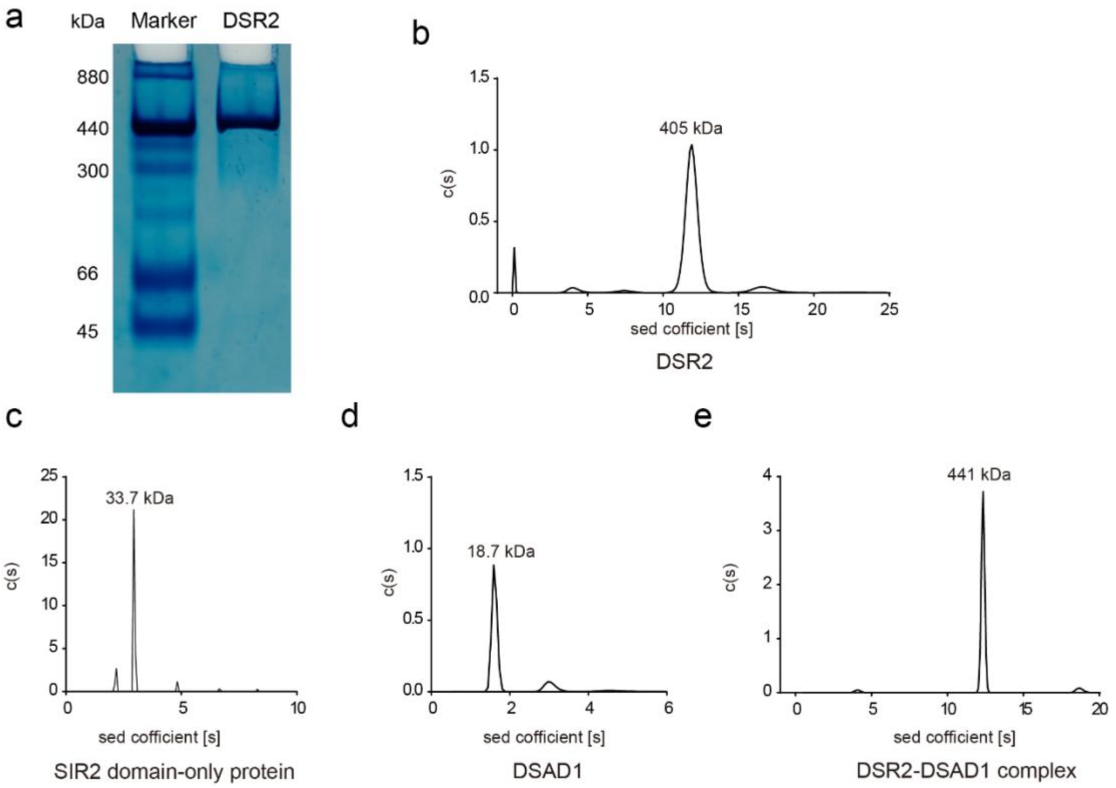

Supplementary Figure 1. DSR2 behaves as a tetramer in solution.

a, Native gel electrophoresis of the full-length DSR2 protein. **b-e,** Analytical ultracentrifugation analysis of the full-length DSR2 protein (**b**), SIR2 domain-only protein (aa 1-303) (**c**), DSAD1 protein (**d**) and DSR2-DSAD1 complex (**e**). Source data are provided as a Source Data file.

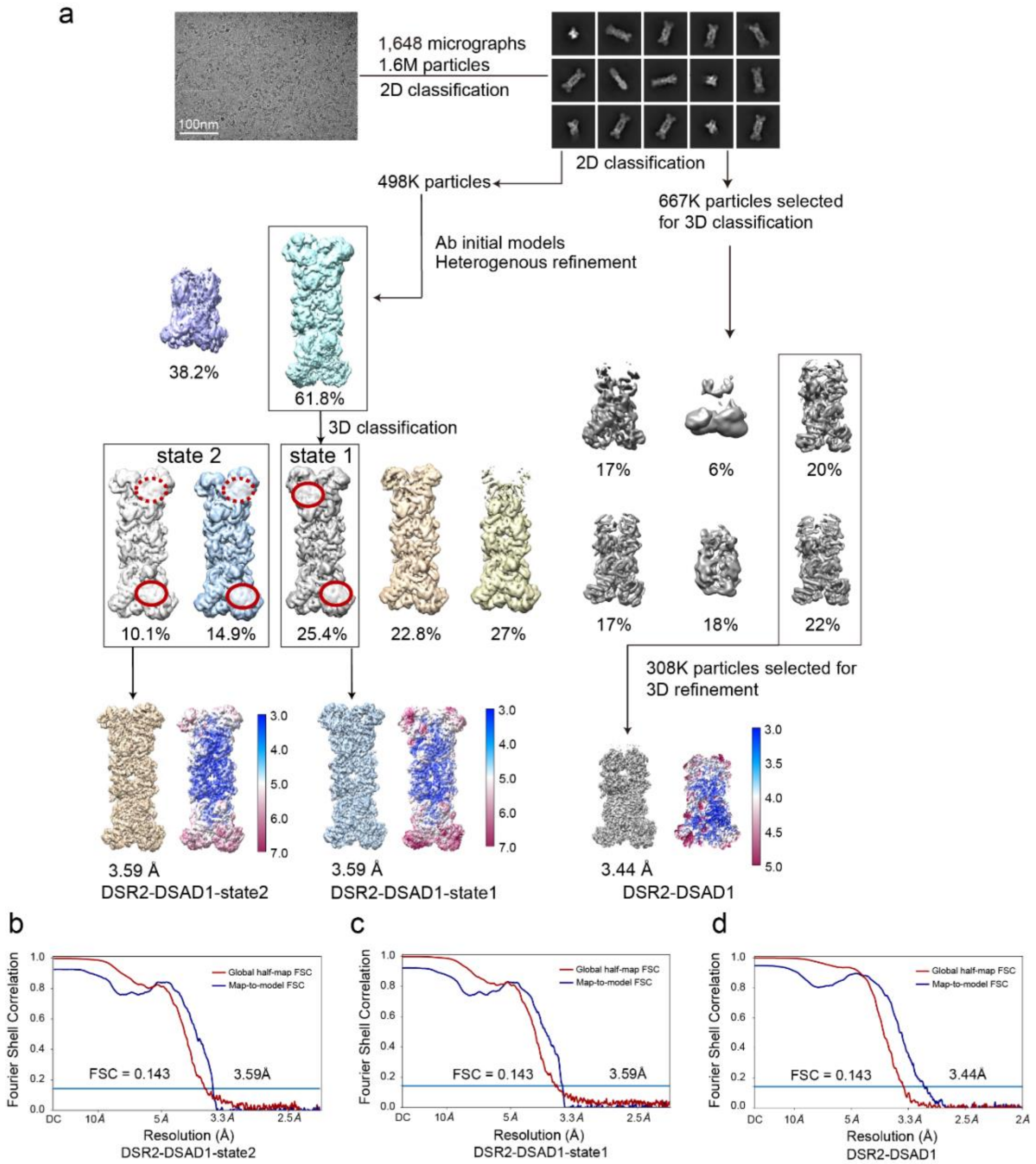

Supplementary Figure 2. Cryo-EM reconstruction of the DSR2-DSAD1 complex. a, A flowchart of cryo-EM data processing for DSR2-DSAD1 complexes with different binding modes. **b-d,** The global half-map FSC (red line), map-to-model FSC (blue line) curves of the reconstructions for the complexes. The resolutions of reconstructions were estimated using the gold-standard cutoff of FSC=0.143.

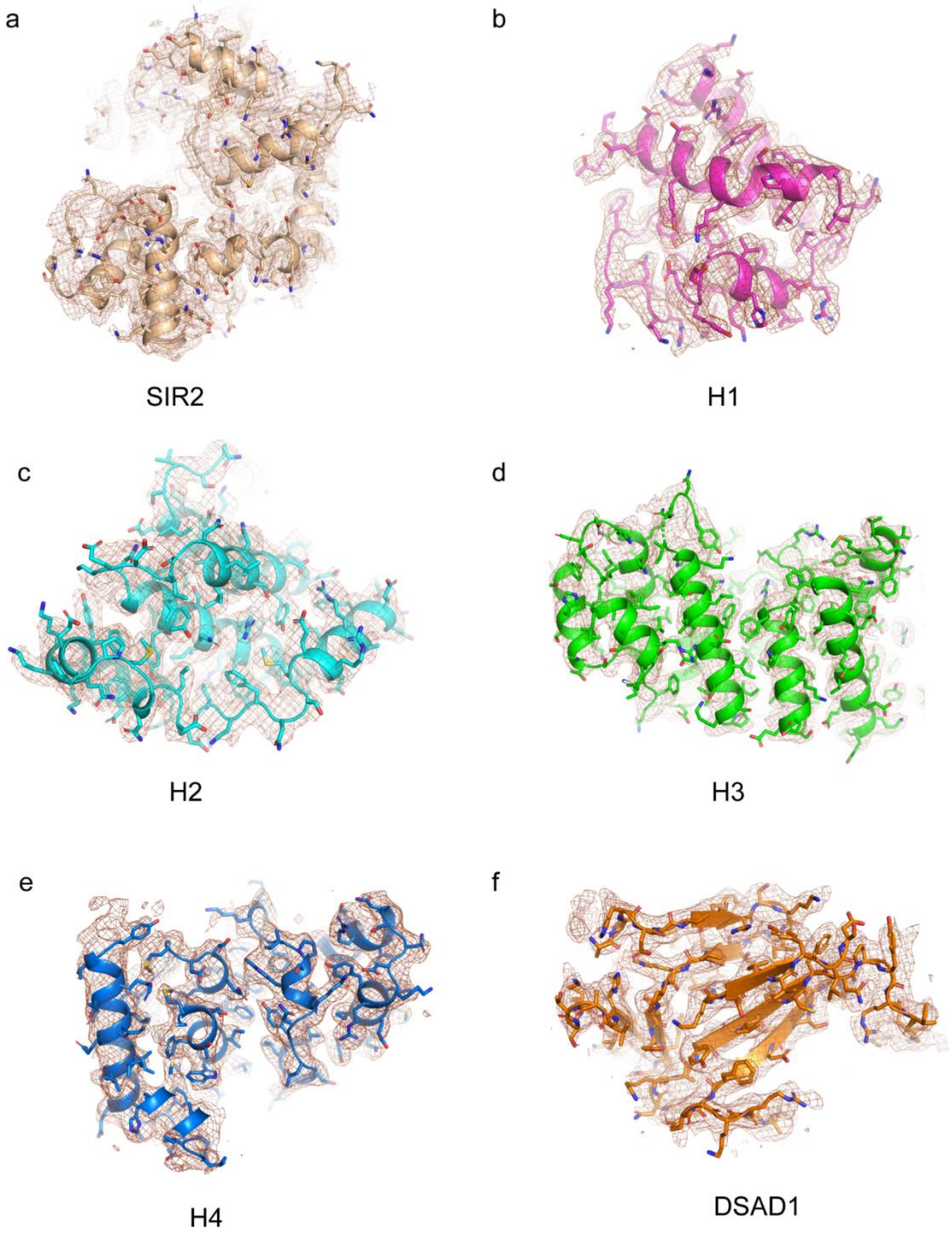

Supplementary Figure 3. Atomic models and the cryo-EM densities of DSR2-DSAD1 complex.

a-f, Fitting of the atomic models to the corresponding EM maps.

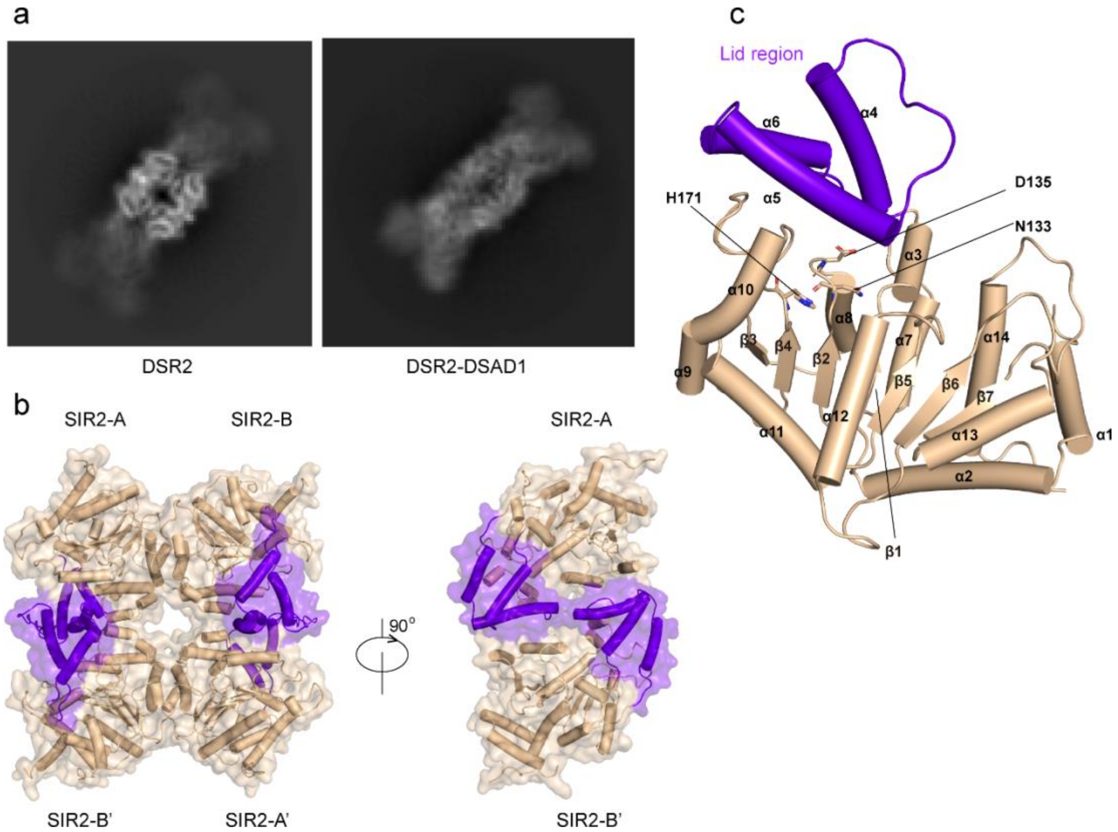

Supplementary Figure 4. Structure of SIR2 domains in the DSR2 tetramer.

a, Representative 2D class averages of DSR2 alone (left) and DSR2-DSAD1 (right, picked from Supplementary Fig. 2a). **b,** The organization of SIR2 domains in the context of DSR2 tetramer. **c,** Close-up view of the SIR2 domain. The Rossmann-fold subdomain and a helical lid subdomain of SIR2 are shown as cartoon and colored in wheat and purple, respectively. The cleft between the two subdomains is thought to be the potential NAD^+ binding site, harboring the catalytic residues Asn133, Asp135 and His171.

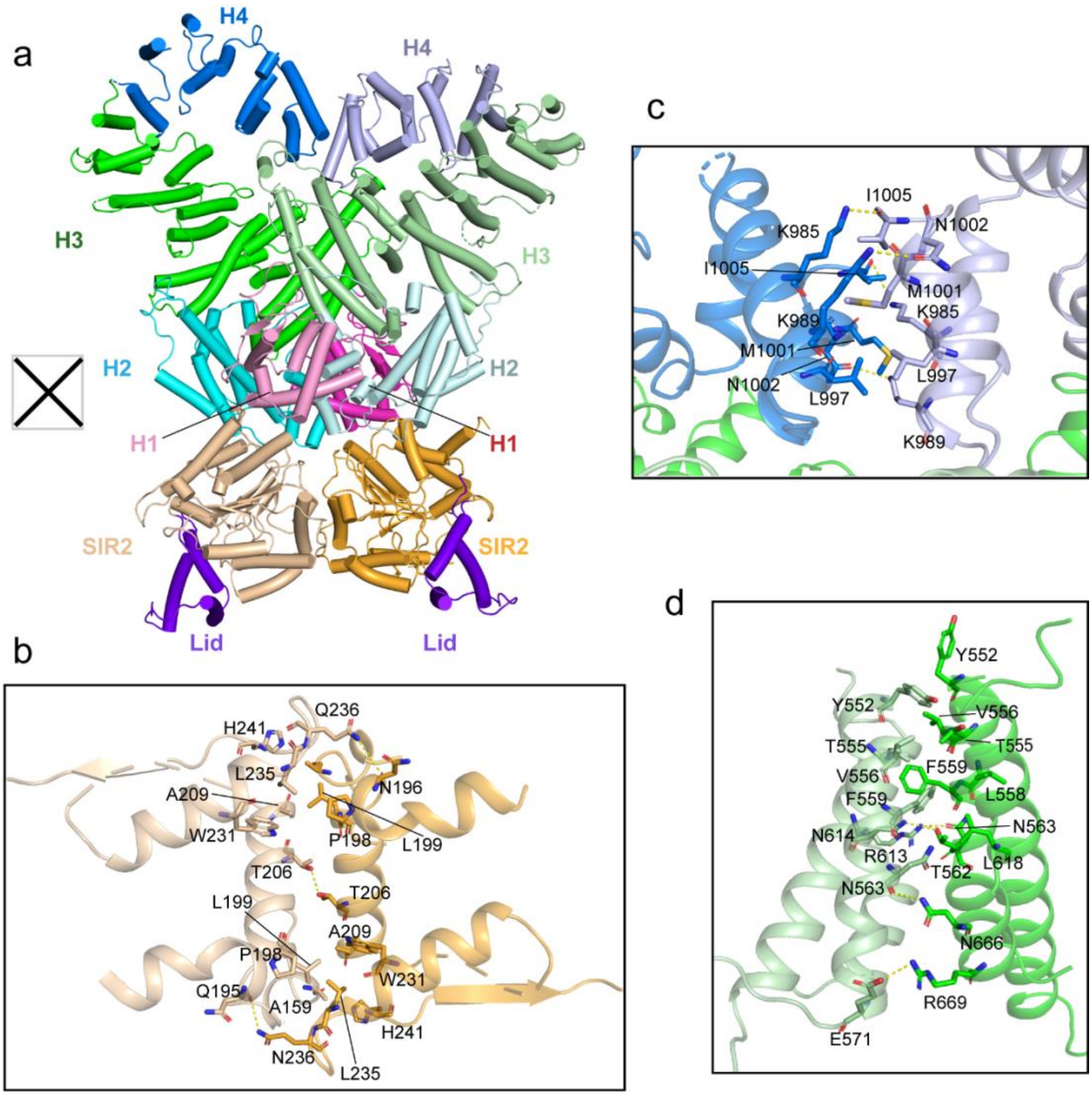

Supplementary Figure 5. The dimerization interface of DSR2.

a, Two DSR2 molecules interact with each other through both the SIR2 and CTD domains, forming an X-shaped architecture. **b,** Close-up view of the SIR2-SIR2 dimerization interface. Key interaction residues in the dimer interface are shown in stick representation. **c,** Two H4 subdomains, located in the throat part of the fishhook-like DSR2, dimerize via hydrophobic and polar contacts. Key interaction residues in the H4 dimerization interface are depicted in stick representation. **d,** Close-up view of the H3 dimerization interface. The residues involved in the H3 dimer interface are shown as sticks.

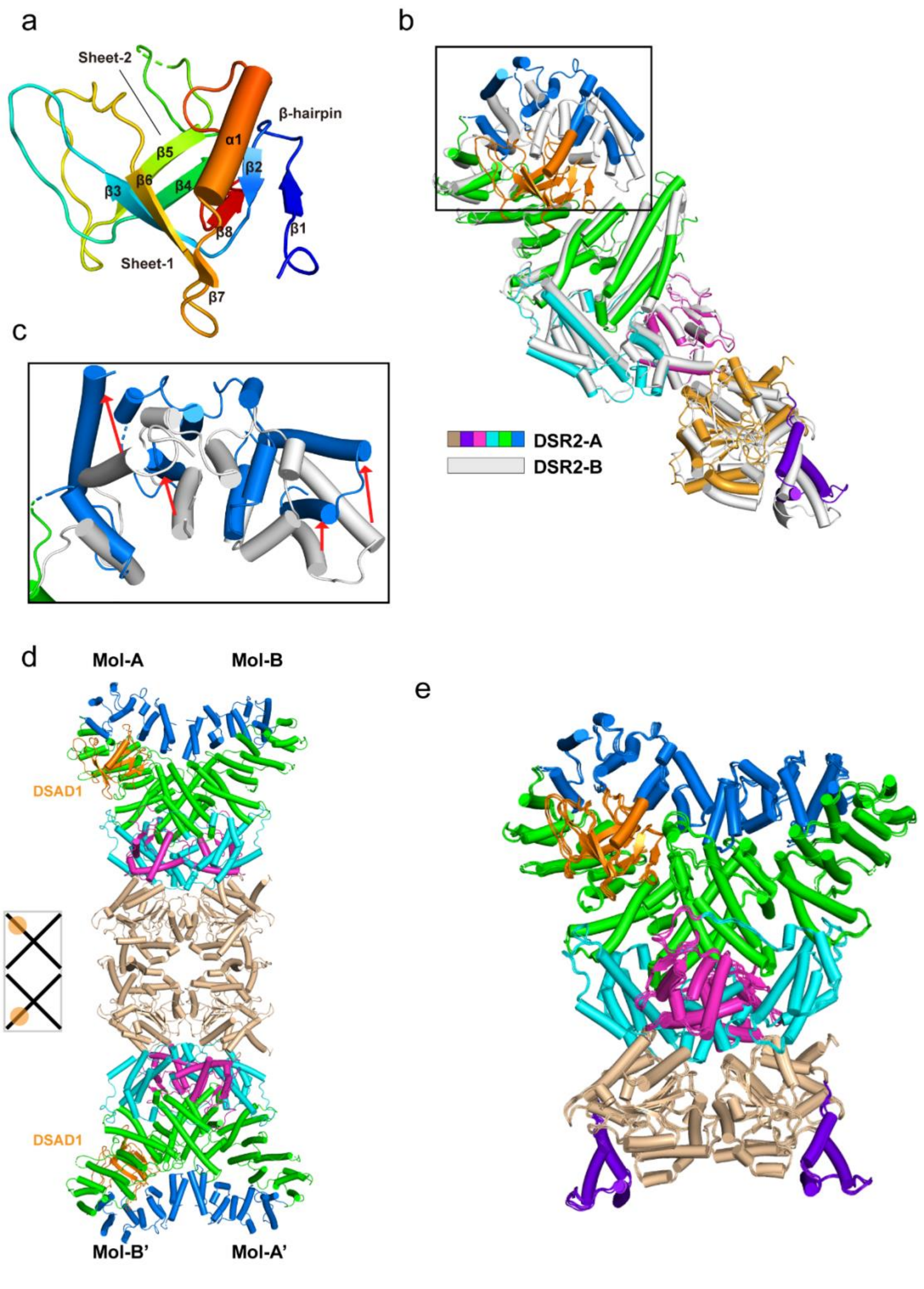

Supplementary Figure 6. Binding of DSAD1 proteins to the DSR2 tetramer.

a, The overall structure of DSAD1 molecule in the DSR2-DSAD1 complex. The DSAD1 is shown in cartoon representation and colored in rainbow. **b,** Structural comparison of the two DSR2 molecules in the presence (DSR2-A) and absence (DSR2-B) of DSAD1. The structural discrepancies observed in the H4 subdomain are marked

with a black box. **c,** The detailed view of the structural discrepancies within the H4 subdomain when aligning the DSAD1-bound DSR2 molecule with the DSAD1-free DSR2 molecule. **d,** Overall structure of DSR2-DSAD1 complex in another binding mode, in which the two DSAD1 molecules bind to the DSR2-A and DSR2-B' molecules, respectively. The black box on the left side shows a schematic of the second binding mode. **e,** Superposition of the DSR2-DSAD1 trimeric subcomplexes in the two binding modes.

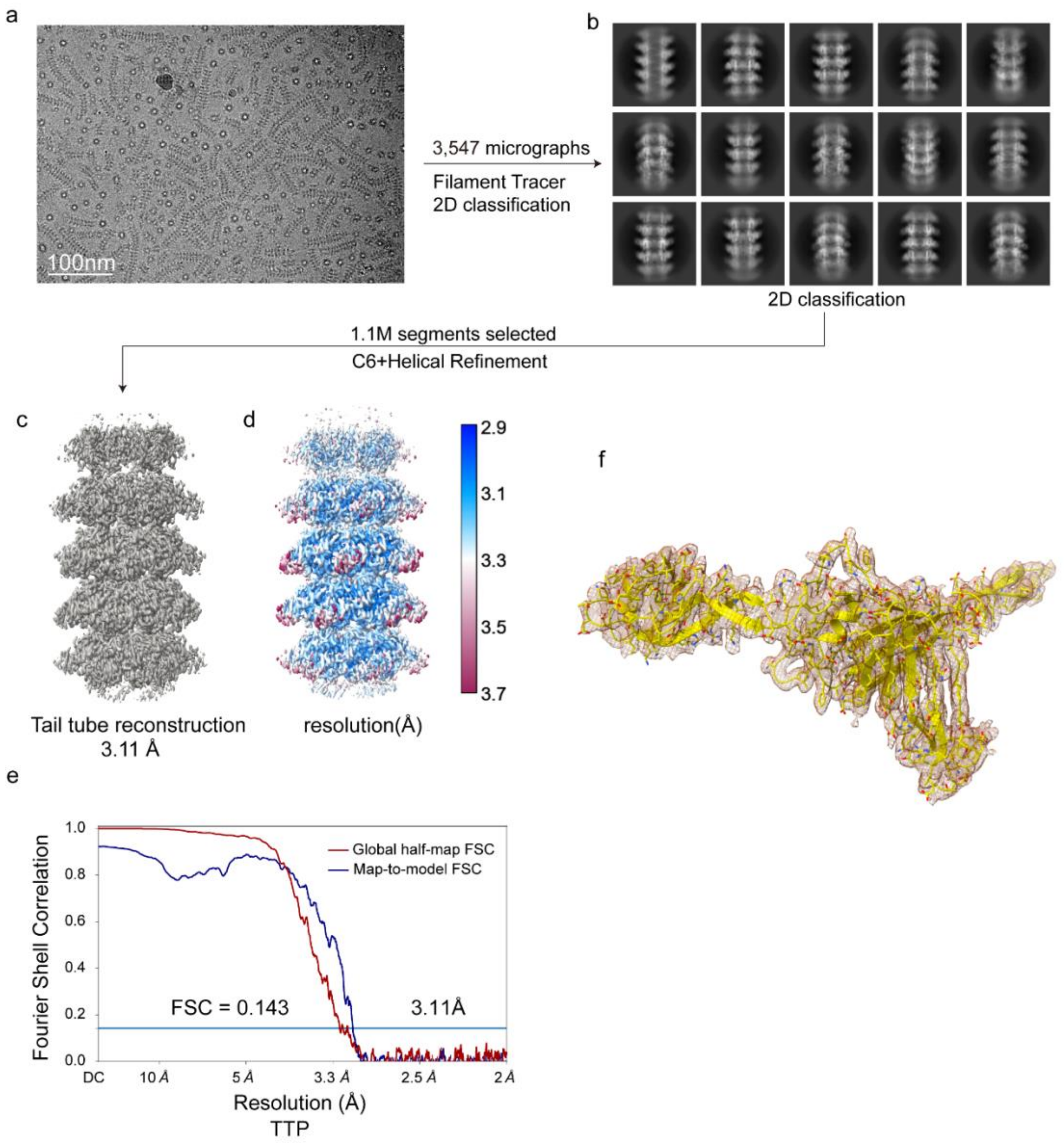

Supplementary Figure 7. Cryo-EM reconstruction of TTP tube.

a, A representative raw cryo-EM micrograph of the TTP tube. **b,** Representative 2D class averages. **c,** 3D reconstruction of Tail tube. **d,** Local resolution of the reconstruction shown in **c**. **e,** The global half-map FSC (red line), map-to-model FSC (blue line) curves of the reconstructions for TTP filament. **f,** Fitting of the atomic model of TTP protein to the corresponding EM map.

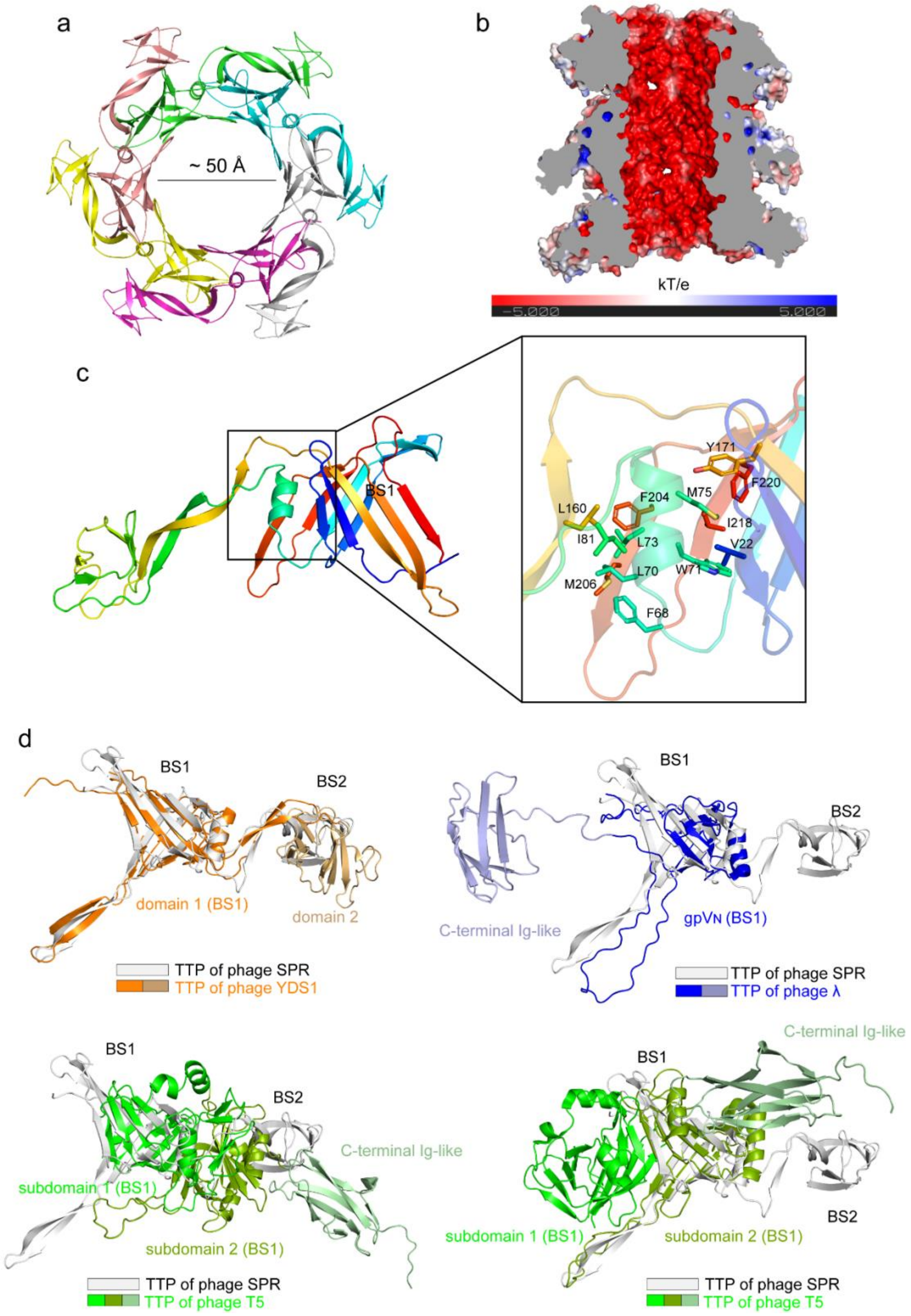

Supplementary Figure 8. The structure features of the TTP tube.

a, A top view of the hexameric TTP rings. Six TTP chains polymerize in a head-to-tail arrangement, forming a ring with a diameter of ~ 50 Å. **b,** Electrostatic surface representation of the TTP tube indicates a negatively charged inner surface. **c,** The

overall structure of the TTP subunit in the cryo-EM structure. The TTP protein is shown as cartoon representations and colored in rainbow (left panel). Close-up view of the intramolecular interface mainly formed by a helix (aa 68-78) (right panel). Key residues involved in the hydrophobic interaction are shown as sticks. **d,** Structural alignment of TTP from phage SPR with those from phages YSD1 (PDB: 6XGR), λ (PDB: 6P3E) and T5 (PDB: 5NGJ). It is noteworthy that the TTP from phage T5 has two similar BS1 regions (subdomains 1 and 2), therefore two structural alignments were made for each BS1 domain. The BS1 core is conserved among *Siphoviridae* phage tails.

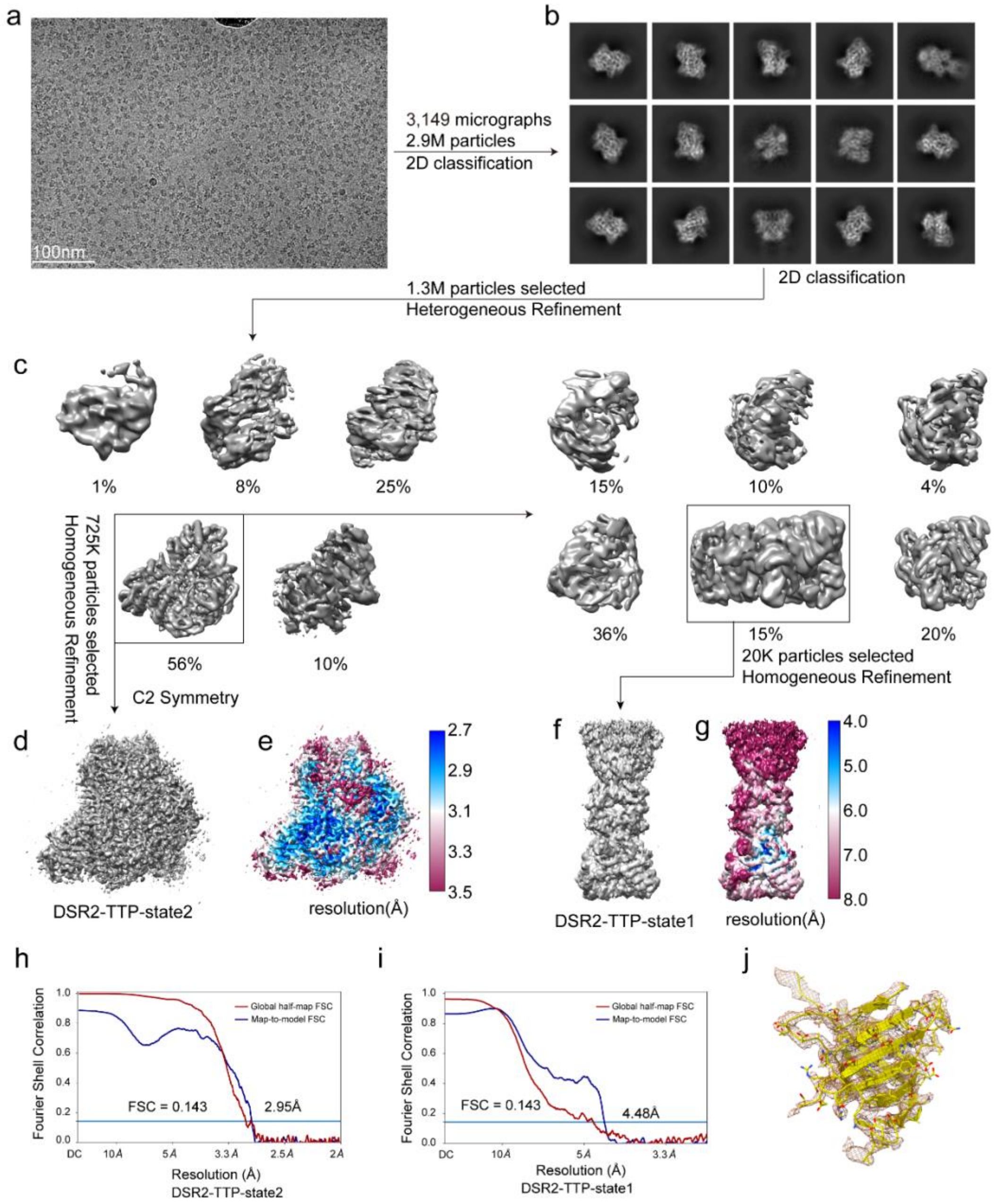

Supplementary Figure 9. Cryo-EM reconstruction of DSR2-TTP complex.

a, A representative raw cryo-EM micrograph of the DSR2-TTP complex. **b,** Representative 2D class averages. **c,** 3D classification. **d,** Final map of DSR2-TTP complex in state 2. **e,** Local resolution of the reconstruction shown in **d**. **f,** Final map of DSR2-TTP complex in state 1. **g,** Local resolution of the reconstruction shown in **f**. **h-i,** Global FSC plot of the reconstruction shown in **d** and **f**. **j,** Fitting of TTP protein in the DSR2-TTP complex to the corresponding EM map.

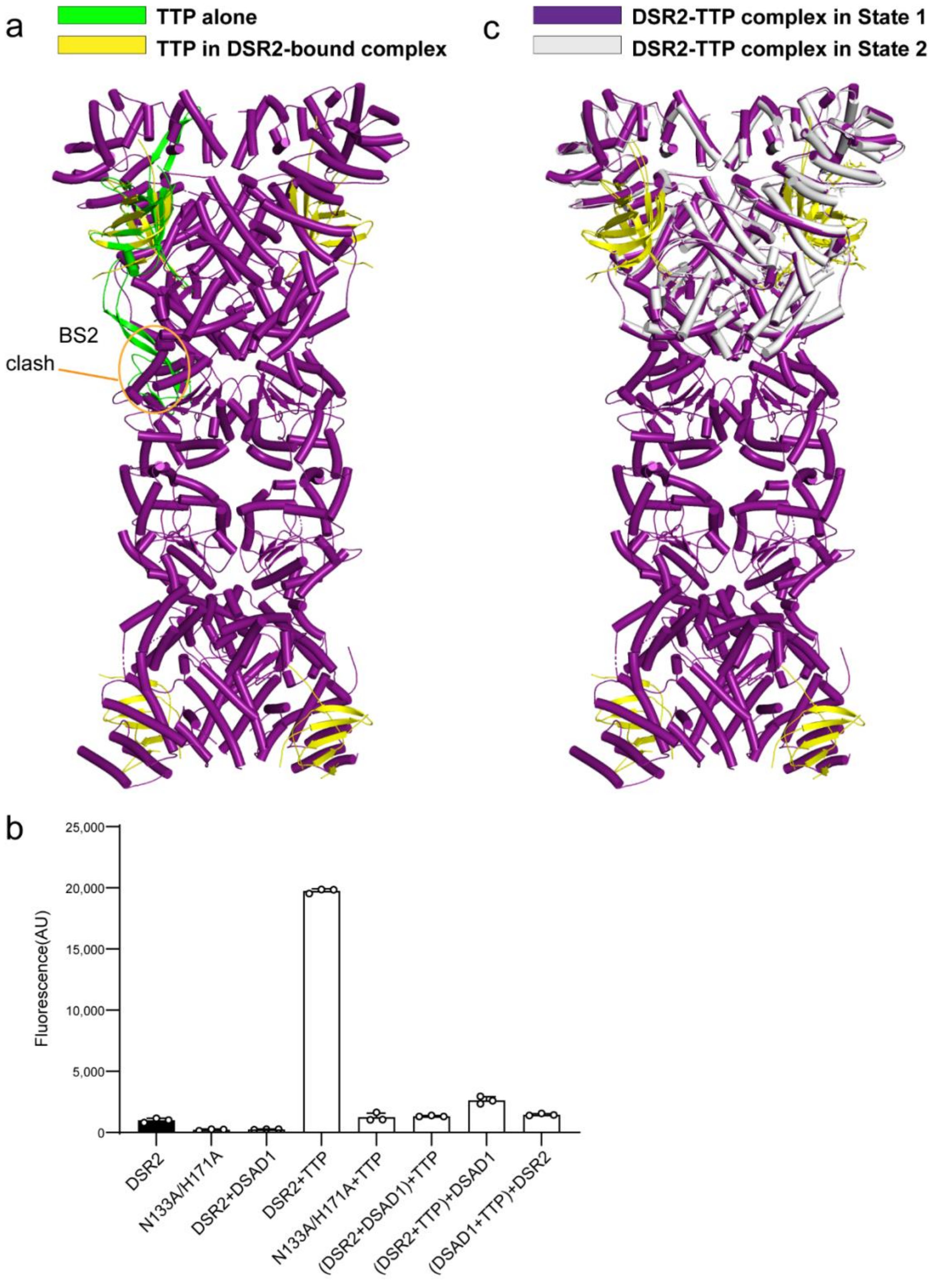

Supplementary Figure 10. DSAD1 and TTP occupy the same binding pocket in DSR2.

a, Structural comparison of TTP units in the two states. The BS2 domain is thought to extended from the TTP-binding pocked, potentially explaining the flexibility of BS2 in the DSR2-bound state. **b,** In vitro NAD^+ degradation assay of DSR2 proteins in the

presence or absence of TTP and DSAD1. All experiment were replicated at least three times (mean ± SD, n=3 independent replicates). **c,** Structural comparison of DSR2-TTP complexes in the two states. The DSR2 molecules in state 1 and state 2 are colored in purple and gray, respectively. The TTP proteins in the two states are colored in yellow.

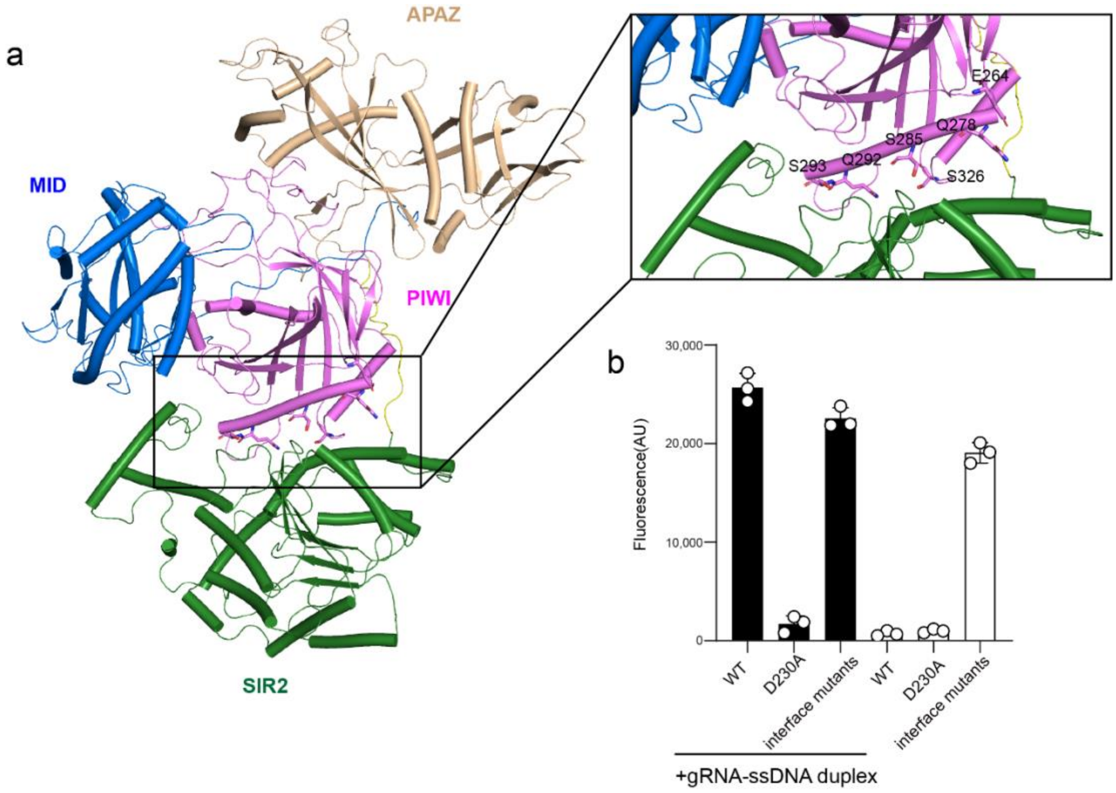

Supplementary Figure 11. Mutation of the residues at the SIR2-PIWI interface of SIR2-APAZ/Ago complex results in active SIR2.

a, The potential interface between SIR2 and PIWI domains is predicted based on the AlphaFold structure.

b, The interface mutant (E264A/Q278A/S285A/Q292A/S293A/S326A) could robustly cleave NAD^+ in the absence of gRNA-ssDNA duplex. All experiment were replicated at least three times (mean ± SD, n=3 independent replicates).

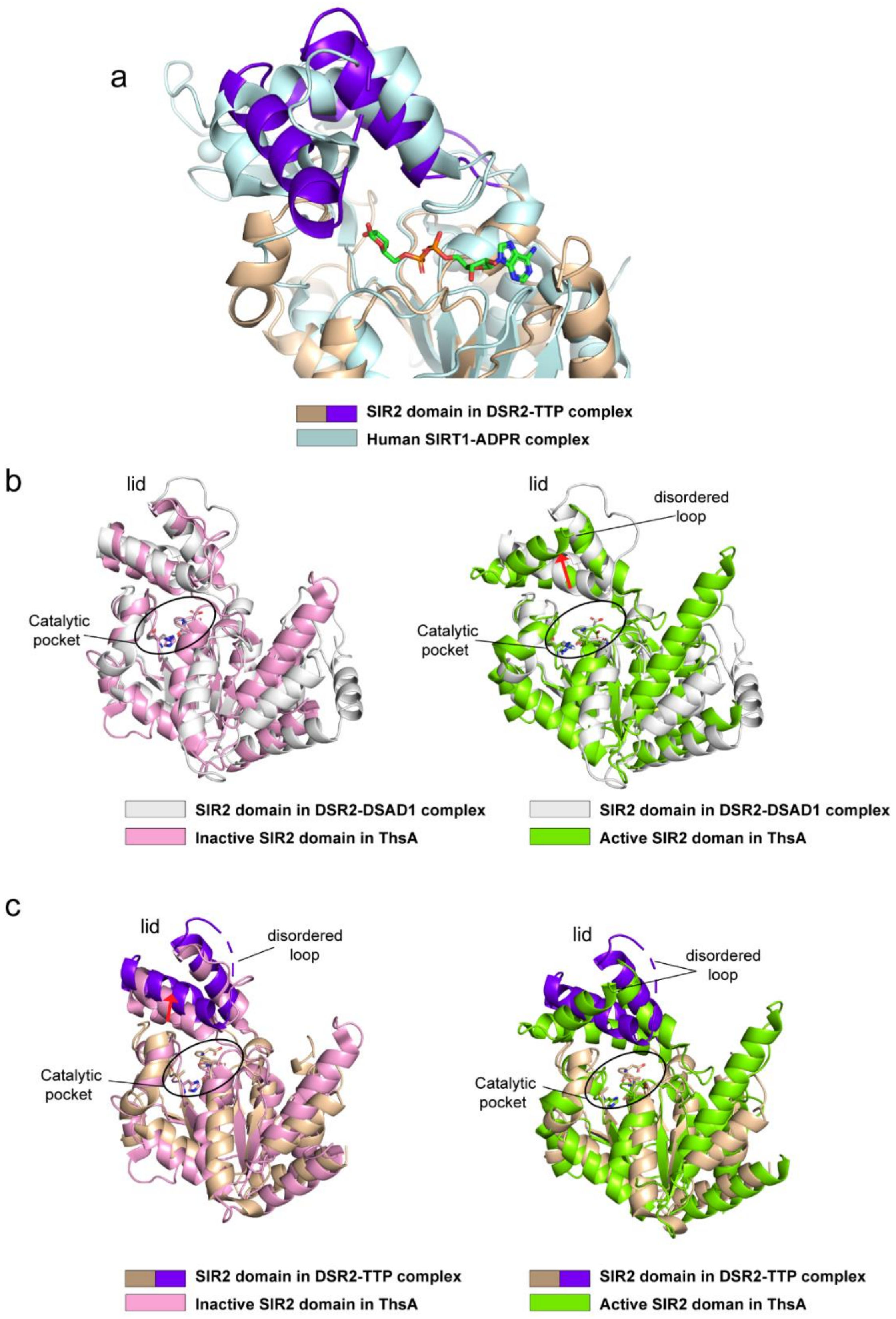

Supplementary Figure 12. Structural comparisons between SIR2 domains.

a, Structural comparison of the SIR2 domain (wheat and purple) in the TTP-bound state and the ADPR-bound SIR2 domain (cyan) in human SIRT1 (PDB ID: 4KXQ). The

ADPR (green) moiety of NAD^+ is shown in stick representation. **b,** Overlaid of the SIR2 domain (white) in the DSAD1-bound state and those in the inactive ThsA structure (pink, PDB ID: 7UXT) or active ThsA structure (green, PDB ID: 6LHX). **c,** Overlaid of the SIR2 domain (wheat and purple) in the TTP-bound state and those in the inactive ThsA structure (pink, PDB ID: 7UXT) or active ThsA structure (green, PDB ID: 6LHX)

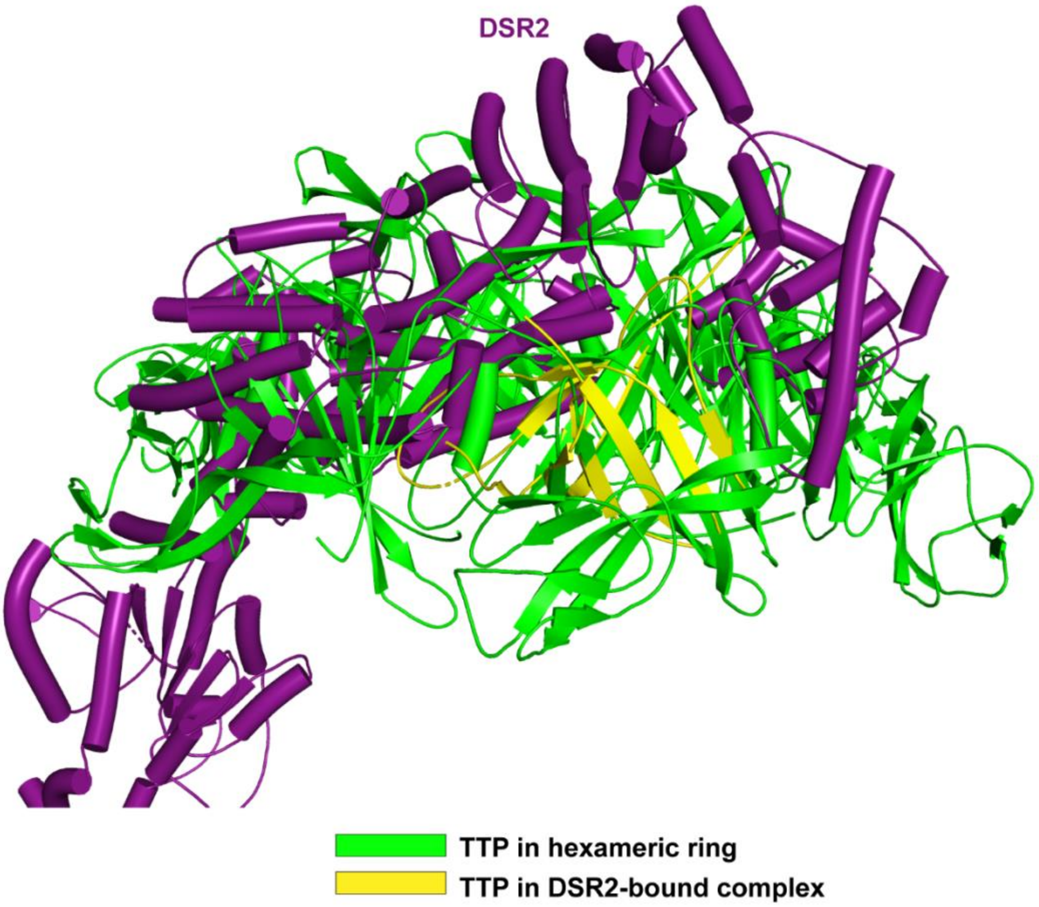

Supplementary Figure 13. Overlaying the TTP in DSR2-bound state onto the TTP in hexameric ring state. Substantial clashes are observed between DSR2 (purple) and TTP in the hexameric ring.

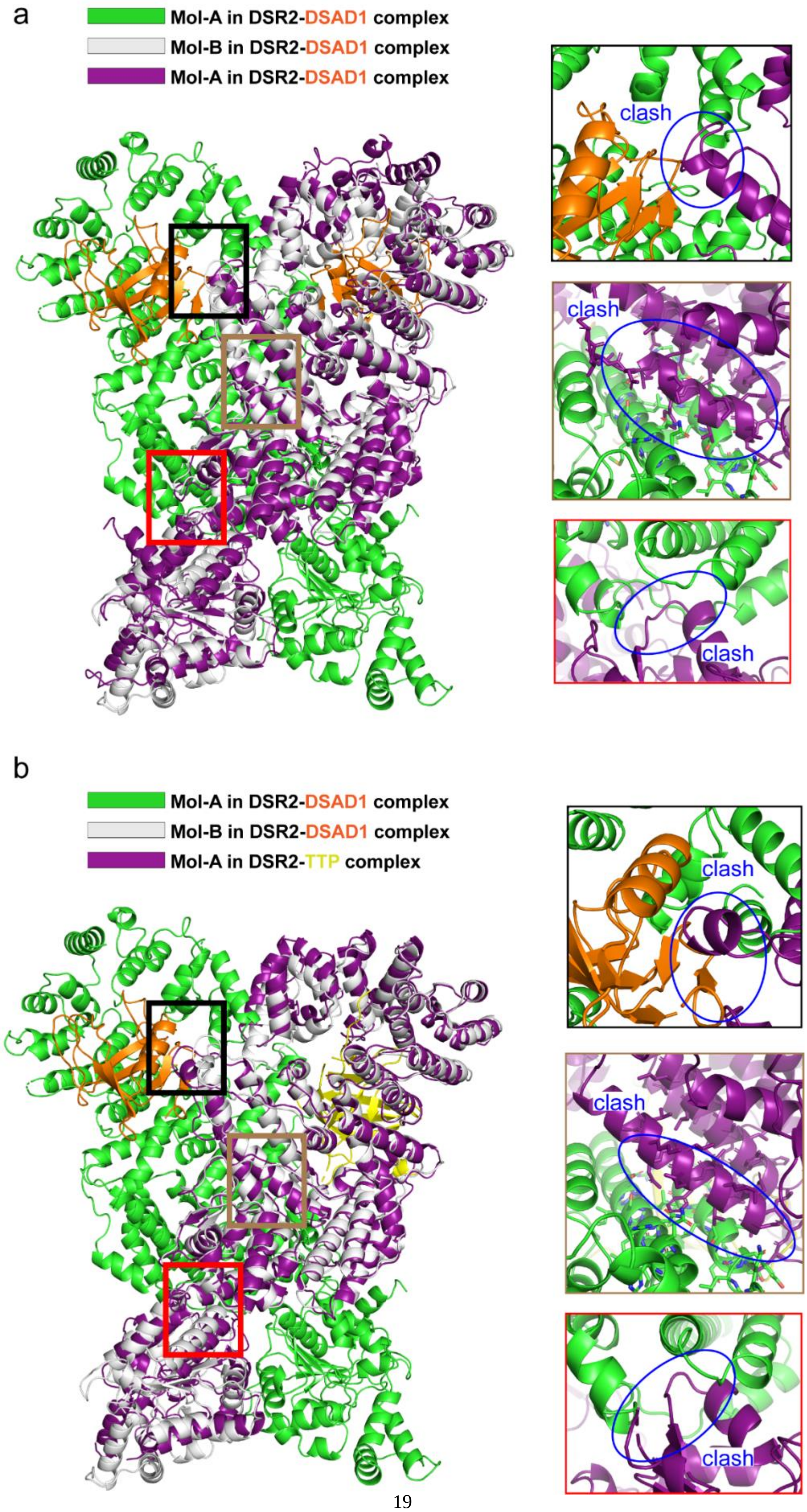
a
Mol-A in DSR2-DSAD1 complex
Mol-B in DSR2-DSAD1 complex
Mol-A in DSR2-DSAD1 complex
clash
clash
clash
b
Mol-A in DSR2-DSAD1 complex
Mol-B in DSR2-DSAD1 complex
Mol-A in DSR2-TTP complex
clash
clash
clash

Supplementary Figure 14. Structural comparisons between ligand-free DSR2 and ligand-bound DSR2.

a, Superposition of the DSAD1-bound DSR2 (Mol-A) and the DSAD1-free DSR2 (Mol-B), and the potential clashes are marked using blue circles. **b,** Docking of the TTP-bound DSR2 (Mol-A) into the DSAD1-free DSR2 (Mol-B), and the potential clashes are marked using blue circles.

Supplementary Table 1. Cryo-EM data collection, refinement and validation statistics.

	DSR2-DSAD1 dataset			TTP dataset	DSR2-TTP dataset	
	DSR2-DSAD1 (EMDB-37603) (PDB 8WKN)	DSR2-DSAD1 state 1 (EMDB-37272) (PDB 8W56)	DSR2-DSAD1 state 2 (EMDB-36982) (PDB 8K9A)	TTP (EMDB-38421) (PDB 8XKN)	DSR2-TTP state 1 (EMDB-37497) (PDB 8WFN)	DSR2-TTP state 2 (EMDB-36980) (PDB 8K98)
**Data collection and processing**						
Magnification	105,000	105,000	105,000	105,000	105,000	105,000
Voltage (kV)	300	300	300	300	300	300
Electron exposure ($e^-/Å^2$)	54	54	54	54	54	54
Defocus range (-μm)	1.2-2.2	1.2-2.2	1.2-2.2	1.2-2.2	1.2-2.2	1.2-2.2
Pixel size (Å)	0.85	0.85	0.85	0.85	0.85	0.85
Symmetry imposed	*C1*	*C1*	*C1*	*Helical & C6*	*C1*	*C2*
Initial particle images (no.)	667,000	498,427	498,427	199,236	2,957,176	2,957,176
Final particle images (no.)	308,000	90.023	96,511	116,902	108,833	725,557
Map resolution (Å)	3.44	3.59	3.59	3.11	4.48	2.95
FSC threshold	0.143	0.143	0.143	0.143	0.143	0.143
Map resolution range (Å)	3.3-5.0	3.4-7.0	3.5-7.0	3.0-5.0	3.8-8.0	2.8-5.0
**Refinement**						
Initial model used	AlphaFold	AlphaFold	AlphaFold	AlphaFold	AlphaFold	AlphaFold
Model resolution (Å)	3.4	3.7	3.8	3.4	4.5	3.2
FSC threshold	0.5	0.5	0.5	0.5	0.5	0.5
Model resolution range (Å)	2.8-3.5	3.5-3.9	3.5-3.9	3.0-3.4	4.2-6.5	2.9-3.3
Map sharpening *B* factor ($Å^2$)	-100	-78.8	-88.4	-140.4	-77.9	-146.2
Model composition						
Non-hydrogen atoms	21527	34091	34074	45888	30004	13415
Protein residues	2607	4110	4106	5808	3613	1619
B factors ($Å^2$)						
Protein						
Min	6.27	45.41	30.46	0.00	126.22	4.73
Max	203.88	355.04	282.61	163.26	564.77	118.43
Mean	101.81	152.03	147.55	81.86	267.45	66.40
R.m.s. deviations						
Bond lengths (Å)	0.003	0.003	0.003	0.005	0.004	0.002
Bond angles (°)	0.551	0.595	0.560	0.588	0.779	0.572
**Validation**						
MolProbity score	1.67	1.72	1.76	1.59	2.38	2.22
Clashscore	6.48	9.79	9.72	5.44	10.24	9.07
Poor rotamers (%)	0.25	0.24	0.29	0.28	4.69	2.67
Ramachandran plot						
Favored (%)	95.47	96.73	96.23	95.68	95.12	93.89
Allowed (%)	4.53	3.24	3.67	4.32	4.74	6.05
Disallowed (%)	0	0.02	0.10	0	0.14	0.06
