# Supplementary material for: Insights into the modulation of bacterial NADase activity by phage proteins
Source: Nat Commun. 2024 Mar 27;15:2692. doi: 10.1038/s41467-024-47030-z (PMC10973363; doi:10.1038/s41467-024-47030-z)
Supplement: Supplementary file 2 — Reporting Summary [file 41467_2024_47030_MOESM2_ESM.pdf]

nature portfolio

Corresponding author(s): Heng Zhang

Last updated by author(s): 2024/3/7

Reporting Summary

Nature Portfolio wishes to improve the reproducibility of the work that we publish. This form provides structure for consistency and transparency in reporting. For further information on Nature Portfolio policies, see our Editorial Policies and the Editorial Policy Checklist.

Statistics

For all statistical analyses, confirm that the following items are present in the figure legend, table legend, main text, or Methods section.

n/a	Confirmed	
☐	☒	The exact sample size (*n*) for each experimental group/condition, given as a discrete number and unit of measurement
☒	☐	A statement on whether measurements were taken from distinct samples or whether the same sample was measured repeatedly
☒	☐	The statistical test(s) used AND whether they are one- or two-sided<br>*Only common tests should be described solely by name; describe more complex techniques in the Methods section.*
☒	☐	A description of all covariates tested
☒	☐	A description of any assumptions or corrections, such as tests of normality and adjustment for multiple comparisons
☐	☒	A full description of the statistical parameters including central tendency (e.g. means) or other basic estimates (e.g. regression coefficient) AND variation (e.g. standard deviation) or associated estimates of uncertainty (e.g. confidence intervals)
☒	☐	For null hypothesis testing, the test statistic (e.g. *F*, *t*, *r*) with confidence intervals, effect sizes, degrees of freedom and *P* value noted<br>*Give P values as exact values whenever suitable.*
☒	☐	For Bayesian analysis, information on the choice of priors and Markov chain Monte Carlo settings
☒	☐	For hierarchical and complex designs, identification of the appropriate level for tests and full reporting of outcomes
☒	☐	Estimates of effect sizes (e.g. Cohen's *d*, Pearson's *r*), indicating how they were calculated

Our web collection on statistics for biologists contains articles on many of the points above.

Software and code

Policy information about availability of computer code

Data collection	Cryo-EM data sets of DSR2-DSAD1 complex, TTP tube and DSR2-TTP complex were collected using a Titan Krios microscope (FEI). Cryo-EM images were collected with EPU at a nominal magnification of 105,000x with a defocus range from –1.2 µm to –2.2 µm and were recorded on a K3 summit electron direct detector in super-resolution mode at the end of a GIF-Quantum energy filter operated with a slit width of 20 eV.
Data analysis	Cryo-EM data processing for the DSR2-DSAD1 complex was performed with cryoSPARC v4.1.0 and RELION-3. Cryo-EM data of TTP tube and DSR2-TTP complex were processed using RELION-3. AlphaFold2 was used to predict initial models of DSR2, TTP and DSAD1 proteins. Model building and refinement were carried out with Coot (version 0.9.7) and phenix.real_space_refine (PHENIX version 1.20.1-4487). Model validation was performed using MolProbity in PHENIX.

For manuscripts utilizing custom algorithms or software that are central to the research but not yet described in published literature, software must be made available to editors and reviewers. We strongly encourage code deposition in a community repository (e.g. GitHub). See the Nature Portfolio guidelines for submitting code & software for further information.

Data

Policy information about availability of data

All manuscripts must include a data availability statement. This statement should provide the following information, where applicable:

- Accession codes, unique identifiers, or web links for publicly available datasets
- A description of any restrictions on data availability
- For clinical datasets or third party data, please ensure that the statement adheres to our policy

The atomic coordinates and EM maps have been deposited in the Protein Data Bank under accession codes 8WKN (DSR2-DSAD1), 8W56 (DSR2-DSAD1 state 1), 8K9A (DSR2-DSAD1 state 2), 8XKN (TTP), 8WFN (DSR2-TTP state 1), 8K98 (DSR2-TTP state 2), and in the Electron Microscopy Data Bank under corresponding accession codes EMD-37603, EMD-37272, EMD-36982, EMD-38421, EMD-37497 and EMD-36980. The TTP structures from phages YSD1 (PDB ID: 6XGR), λ (PDB ID: 6P3E) and T5 (PDB ID: 5NGJ), human SIRT1 structure (PDB ID: 4KXQ), ThsA structures (PDB IDs: 7UXT and 6LHX) were used for structural comparison.

Research involving human participants, their data, or biological material

Policy information about studies with human participants or human data. See also policy information about sex, gender (identity/presentation), and sexual orientation and race, ethnicity and racism.

Reporting on sex and gender	N/A
Reporting on race, ethnicity, or other socially relevant groupings	N/A
Population characteristics	N/A
Recruitment	N/A
Ethics oversight	N/A

Note that full information on the approval of the study protocol must also be provided in the manuscript.

Field-specific reporting

Please select the one below that is the best fit for your research. If you are not sure, read the appropriate sections before making your selection.

☒ Life sciences ☐ Behavioural & social sciences ☐ Ecological, evolutionary & environmental sciences

For a reference copy of the document with all sections, see nature.com/documents/nr-reporting-summary-flat.pdf

Life sciences study design

All studies must disclose on these points even when the disclosure is negative.

Sample size	At least three independent replicates of each in vitro NAD degradation assay were performed. The error bars represent the standard deviations. Each pull-down assay in the manuscript was performed three times, and the gels shown in the figures represent three independent replicate experiments.
Data exclusions	During cryo-EM structure determination, poor-quality images and particles were excluded following standard procedures of RELION-3 or cryoSPARC. Detailed information regarding the number of particles used in the cryo-EM structure determination is provided in the methods section of the manuscript and supplementary figures.
Replication	The in vitro NAD degradation assays and pull-down assays performed in this study were replicated a minimum of three times. Detailed information about the replication can be found in the figure legends and methods section.
Randomization	Not relevant
Blinding	N/A

Reporting for specific materials, systems and methods

We require information from authors about some types of materials, experimental systems and methods used in many studies. Here, indicate whether each material, system or method listed is relevant to your study. If you are not sure if a list item applies to your research, read the appropriate section before selecting a response.

Materials & experimental systems

n/a	Involved in the study
☒	☐ Antibodies
☒	☐ Eukaryotic cell lines
☒	☐ Palaeontology and archaeology
☒	☐ Animals and other organisms
☒	☐ Clinical data
☒	☐ Dual use research of concern
☒	☐ Plants

Methods

n/a	Involved in the study
☒	☐ ChIP-seq
☒	☐ Flow cytometry
☒	☐ MRI-based neuroimaging

Plants

Seed stocks	N/A
Novel plant genotypes	N/A
Authentication	N/A
